# Targeted germ line disruptions reveal general and species-specific roles for paralog group 1 *hox* genes in zebrafish

**DOI:** 10.1186/1471-213X-14-25

**Published:** 2014-06-05

**Authors:** Steven E Weicksel, Ankit Gupta, Denise A Zannino, Scot A Wolfe, Charles G Sagerström

**Affiliations:** 1 Department of Biochemistry and Molecular Pharmacology, University of Massachusetts Medical School, 364 Plantation St./LRB815, Worcester, MA 01605-2324, USA; 2 Program in Gene Function and Expression, University of Massachusetts Medical School, Worcester, MA 01605, USA

**Keywords:** Zinc finger nuclease, TALEN nuclease, Retinoic acid signaling, Hindbrain, Nucleosome positioning, Gene expression

## Abstract

**Background:**

The developing vertebrate hindbrain is transiently segmented into rhombomeres by a process requiring *Hox* activity. *Hox* genes control specification of rhombomere fates, as well as the stereotypic differentiation of rhombomere-specific neuronal populations. Accordingly, germ line disruption of the paralog group 1 (PG1) *Hox* genes *Hoxa1* and *Hoxb1* causes defects in hindbrain segmentation and neuron formation in mice. However, antisense-mediated interference with zebrafish *hoxb1a* and *hoxb1b* (analogous to murine *Hoxb1* and *Hoxa1*, respectively) produces phenotypes that are qualitatively and quantitatively distinct from those observed in the mouse. This suggests that PG1 *Hox* genes may have species-specific functions, or that anti-sense mediated interference may not completely inactivate *Hox* function in zebrafish.

**Results:**

Using zinc finger and TALEN technologies, we disrupted *hoxb1a* and *hoxb1b* in the zebrafish germ line to establish mutant lines for each gene. We find that zebrafish *hoxb1a* germ line mutants have a more severe phenotype than reported for Hoxb1a antisense treatment. This phenotype is similar to that observed in *Hoxb1* knock out mice, suggesting that *Hoxb1/hoxb1a* have the same function in both species. Zebrafish *hoxb1b* germ line mutants also have a more severe phenotype than reported for *hoxb1b* antisense treatment (e.g. in the effect on Mauthner neuron differentiation), but this phenotype differs from that observed in *Hoxa1* knock out mice (e.g. in the specification of rhombomere 5 (r5) and r6), suggesting that *Hoxa1/hoxb1b* have species-specific activities. We also demonstrate that Hoxb1b regulates nucleosome organization at the *hoxb1a* promoter and that retinoic acid acts independently of *hoxb1b* to activate *hoxb1a* expression.

**Conclusions:**

We generated several novel germ line mutants for zebrafish *hoxb1a* and *hoxb1b.* Our analyses indicate that *Hoxb1* and *hoxb1a* have comparable functions in zebrafish and mouse, suggesting a conserved function for these genes. In contrast, while *Hoxa1* and *hoxb1b* share functions in the formation of r3 and r4, they differ with regards to r5 and r6, where *Hoxa1* appears to control formation of r5, but not r6, in the mouse, whereas *hoxb1b* regulates formation of r6, but not r5, in zebrafish. Lastly, our data reveal independent regulation of *hoxb1a* expression by retinoic acid and Hoxb1b in zebrafish.

## Background

*Hox* genes encode a conserved family of homeodomain-containing transcription factors essential for metazoan development [1-4]. As a result of duplication events, vertebrate genomes contain four clusters of *Hox* genes, with the exception of teleost fish that have undergone an additional genome duplication - for instance, the zebrafish genome contains seven *Hox* clusters [4]. In most cases, genes that occupy the same position in different clusters (known as paralogs) have similar expression patterns and functions, leading to redundancy of *Hox* function. During early development, *Hox* genes specify tissue identities along the anterior-posterior (AP) axis of the animal. The linear arrangement of *Hox* genes in the genomic clusters coincides with the timing and position of their expression along the AP axis, a characteristic termed colinearity [3,5,6]. The retinoic acid (RA) signaling pathway activates early *Hox* gene expression and is important in colinear regulation [7,8]. RA binds a heterodimeric complex of RA receptors (RARs) and retinoic X receptors (RXRs) that target cis-regulatory sites known as RA response elements (RAREs) in the *Hox* clusters [9-11]. RA promotes decondensation of *Hox* clusters from compact chromosomal chromatin in cells and embryos [12-14] and this process correlates with the progressive activation of *Hox* transcription along the genomic cluster. Once transcribed, *Hox* genes also regulate the expression of other *Hox* genes in auto- and cross-regulatory loops. The highly conserved process of *Hox* gene activation and regulation leads to an overlapping series of *Hox* expression domains along the AP axis, sometimes referred to as the “*Hox* code” [15].

During early embryogenesis, the presumptive vertebrate hindbrain is transiently divided into seven to eight segments (rhombomeres) and *Hox* genes play a key role in formation of the more posterior rhombomeres [16]. Each rhombomere gives rise to unique cell populations from which segment-specific motor neurons and reticulospinal neurons differentiate. For motor neurons, this includes the trigeminal neurons in rhombomere 2 (r2) and r3, the facial motor neurons (FMNs) in r4, the abducens neurons in r5 and r6, and the vagal neurons in the caudal hindbrain. These neuronal pools form the motor neuron nuclei of the V^th^, VI^th^, VII^th^ and X^th^ cranial nerves that innervate the face, head, and neck of the animal. Reticulospinal neurons also form in a rhombomere determinate manner and regulate breathing and circulation, as well as the coordination of locomotor signals between the spinal cord and the brain. For instance, the Mauthner neurons (MNs) form in r4 and extend axons contralaterally and posteriorly and function to control the escape response. Segmentation of the hindbrain starts with the formation of r4 followed by r1/r2, r3, r7, and r5/r6 [17]. Accordingly, the first *Hox* genes transcribed in the mouse, *Hoxa1* and *Hoxb1,* are expressed in r4 [5,18,19]. A series of loss-of-function studies have determined that *Hoxa1* and its downstream target *Hoxb1* have separate functions. In particular, *Hoxa1* mutant mouse embryos have segmentation defects while *Hoxb1* mutants appear to have neuronal defects related to r4 specification. The segmentation defects observed in *Hoxa1* mutants include an enlarged r3, a reduced r4, and a reduced or completely lost r5 [20-24]. Similar segmentation defects are also found in mice with mutations made to the retinoic acid response element (RARE) found in the downstream enhancer of *Hoxa1*[25]. These segmentation defects are specific to the function of *Hoxa1,* as mouse *Hoxb1* mutant embryos show no defects in hindbrain segmentation [26,27]. While hindbrain segments form normally in *Hoxb1* mutants, r4 derived FMNs fail to migrate into r5 [23,26,28] and instead migrate away from the midline of r4, assuming lateral positions similar to trigeminal neurons in r3. FMN neurons in *Hoxb1* mutants extend axons out through r4 into the second pharyngeal arch similar to the projection of wild type FMN axons. Loss-of-function studies for zebrafish *hoxb1b* and *hoxb1a* (functionally analogous to murine *Hoxa1* and *Hoxb1*, respectively) have been accomplished using antisense morpholino oligos (MOs) to block translation of *hoxb1b* and *hoxb1a*. Embryos injected with *hoxb1bMO* have hindbrain segmentation defects with an expanded r3 and a reduced r4, r5 and r6, while hindbrain segmentation is unaffected in *hoxb1aMO-*injected embryos [29]. Furthermore, *hoxb1aMO*-injected embryos possess FMNs remaining in r4 that resemble the stalled FMNs observed in mouse *Hoxb1* mutants. While these data indicate that the zebrafish *hoxb1b* and *hoxb1a* genes have roles similar to those of mouse *Hoxa1* and *Hoxb1*, there are also differences between the defects observed in the mouse mutants versus MO-injected zebrafish. First, segmentation defects appear more severe in *Hoxa1*^*−/−*^ and *Hoxa1*^*−/−*^*;Hoxb1*^*−/−*^ mutants than in *hoxb1bMO* and *hoxb1bMO;hoxb1aMO*-injected zebrafish embryos. Specifically, the r5 domain in *Hoxa1*^*−/−*^ mouse embryos is lost, while r5 is merely reduced in zebrafish *hoxb1bMO*-injected embryos. *Hoxa1*^*−/−*^*;Hoxb1*^*−/−*^ mice also appear to have a stronger segmentation defect, with loss of both r4 and r5, while *hoxb1bMO;hoxb1aMO*-injected zebrafish embryos show only a 50% reduction in the size of r4. Second, unlike the *Hoxa1*^*−/−*^ mice, *hoxb1bMO*-injected embryos have a reduced r6. Third, the *hoxb1aMO* shows no effect on the reticulospinal neurons in r4 although *Hoxb1* mutant mice have a miss-specified r4. Indeed, even when *hoxb1bMO* and *hoxb1aMO* are co-injected, only incomplete formation of Mauthner neurons is observed in r4. These phenotypic differences suggest that either these genes function differently in mouse and zebrafish, or the MO phenotypes do not recapitulate true loss-of-function of *hoxb1b* and *hoxb1a*. Here we report the generation of *hoxb1a* and *hoxb1b* germ line mutations using zinc finger and TALE nucleases in zebrafish. We find that these germ line mutants have more severe phenotypes than those reported from antisense MO injections, suggesting that the MOs do not completely block *Hox* function. Our results demonstrate that *hoxb1a* is required for specification of r4 and for the formation of r4-specific neurons. This is similar to the reported mouse phenotype, suggesting that *hoxb1a* and *Hoxb1* share conserved functions in r4 formation. Similarly, our results reveal that zebrafish *hoxb1b* and mouse *Hoxa1* share functions in the formation of r3 and r4, but their roles also differ, such that *Hoxa1* is required in mouse r5 while *hoxb1b* is required in zebrafish r6. Lastly, we demonstrate that *hoxb1b* and retinoic acid act separately to activate *hoxb1a* expression, with Hoxb1b modulating nucleosome organization at the *hoxb1a* promoter.

## Results

### Generation of *hoxb1a* and *hoxb1b* germ line mutants

To investigate the roles of *hoxb1a* and *hoxb1b* in zebrafish hindbrain development, we generated *hoxb1a* and *hoxb1b* loss of function mutants using zinc finger nucleases (ZFNs) and TALE nucleases (TALENs). ZFNs and TALENs consist of the Fok1 endonuclease tethered to sequence-specific DNA-binding domains (zinc finger or TALE) that target the nuclease to a desired genomic location [30,31]. The use of obligate heterodimeric FokI nucleases increases target specificity by requiring that two complementary FokI nuclease domains bind to adjacent genomic sequences for activity [32,33], thereby reducing, but not eliminating, the likelihood of off-target effects. Once activated through dimerization, the Fok1 nuclease introduces a double strand DNA break that is repaired through the non-homologous end joining (NHEJ) repair pathway. NHEJ is error prone and will introduce mutations at a low rate. While many of the resulting mutations do not affect protein function, we were particularly interested in identifying the small number of mutations that lead to shifts in the reading frame and introduce stop codons.

We initially employed ZFNs to target both *hoxb1a* and *hoxb1b* based on several criteria. First, nucleases were targeted to a site in the first exon of each gene in order to increase the likelihood that a frame shift would terminate translation upstream of known functional domains, particularly the homeodomain. Second, the spacing between the target sequences for each ZFN pair was set to either 5 bp or 6 bp based on previous reports indicating that these represent optimal spacing [34]. Third, we targeted regions containing a restriction site that could be used to screen for mutations. Based on these criteria, we designed several ZFNs to each gene using three separate ZFN “builds” (Table 1). The first and second builds were based on a modular library of single zinc finger proteins [35] . For build 1, we generated one ZFN targeting *hoxb1a* (Zb1a-1) and one targeting *hoxb1b* (Zb1b-1) where each ZFN contained three zinc fingers (ZFs) assembled from the modular library. Build 2 (Zb1a-2 and Zb1b-2) employed the same modular library and targeted the same genomic sites as build 1, but each fusion protein contained four ZFs to increase specificity for the target sequence while decreasing the instances of off-target effects. For build 3, we designed two ZFNs to each gene (Zb1a-3, Zb1a-4, Zb1b-3 and Zb1b-4). Zb1b-3 and Zb1b-4 targeted the same genomic sequence as Zb1b-1 and Zb1b-2, although Zb1b-4 was offset 3 bp relative to Zb1b-3, while Zb1a-3 and Zb1a-4 targeted sites 60 bp and 125 bp, respectively, upstream of the site targeted by Zb1a-1 and Zb1a-2. The ZFNs designed in build 3 also used four ZFs each, but were assembled from an updated version of the ZF library that includes two-finger modules [36]. These two-finger modules were optimized for their ability to bind DNA efficiently in tandem to minimize the impact of context-dependent effects on recognition when fingers are assembled into arrays.

**Table 1:** **Characteristics of TALE and zinc finger nucleases targeting *hoxb1a *** **and *hoxb1b***

**TALEN/ZFN**	**Target coordinate**^**a**^	**Upstream TALEN/ZFN**	**Downstream TALEN/ZFN**	**Gap**^**c**^	**No. of units**^**d**^	**Embryos injected**^**e**^	**Activity**^**f**^	**Diagnostic enzyme**^**g**^
		**Target sequence**^**b**^	**Target sequence**^**b**^					
Zb1b-1	Chr12:28712770	GGTGGAAGG	GTGGACATG	5bp	3	274	NO	BslI
Zb1b-2	Chr12:28712770	GGTGGAAGGGCT	GTGGACATGGGT	5bp	4	424	NO	BslI
Zb1b-3	Chr12:28712770	GTGGAAGGGCTG	GTGGACATGGGT	6bp	4	109	YES	BslI
Zb1b-4	Chr12:28712773	GAGGTGGAAGGG	GACATGGGTAAA	6bp	4	149	YES	BslI
Zb1a-1	Chr3:24060660	GCTGATAAG	GATGCGAAG	6bp	3	2916	NO	FatI
Zb1a-2	Chr3:24060660	GCTGATAAGATG	GATGCGAAGGCC	6bp	4	541	NO	FatI
Zb1a-3	Chr3:24060602	GCCATAGTGTGG	GCCGGTGCGTAC	6bp	4	72	NO	BslI
Zb1a-4	Chr3:24060535	AGGGTTGATAAA	GGATGGGATGTA	5 p	4	56	NO	BslI
Tb1a-1	Chr3:24060209	TCCAGAATGAACTC	TCCCACGGTTACAAAT	16bp	15/16	50	NO	RsaI
Tb1a-2	Chr3:24060227	CTTGGAGTACACAAT	TGGGCGAGTAGGCGTT	16bp	16/16	50	YES	BtgI
Tb1a-3	Chr3:24060213	CCAGAATGAACTCTTTC	TCGTCCCACGGTTAC	16bp	18/15	50	NO	RsaI

^a^Target coordinate as defined by midpoint between the upstream and downstream nuclease recognition sites.

^b^Genomic sequence targeted by the upstream and downstream TALE and ZF nucleases.

^c^Distance between upstream and downstream TALE and ZF nucleases.

^d^The number of TAL or ZF units used to assemble each nuclease.

^e^The number of embryos injected to test activity and raise mutant families.

^f^Nucleases that disrupted the diagnostic restriction site were defined as active.

^g^The enzyme used to test activity of nucleases.

**Figure 1:**
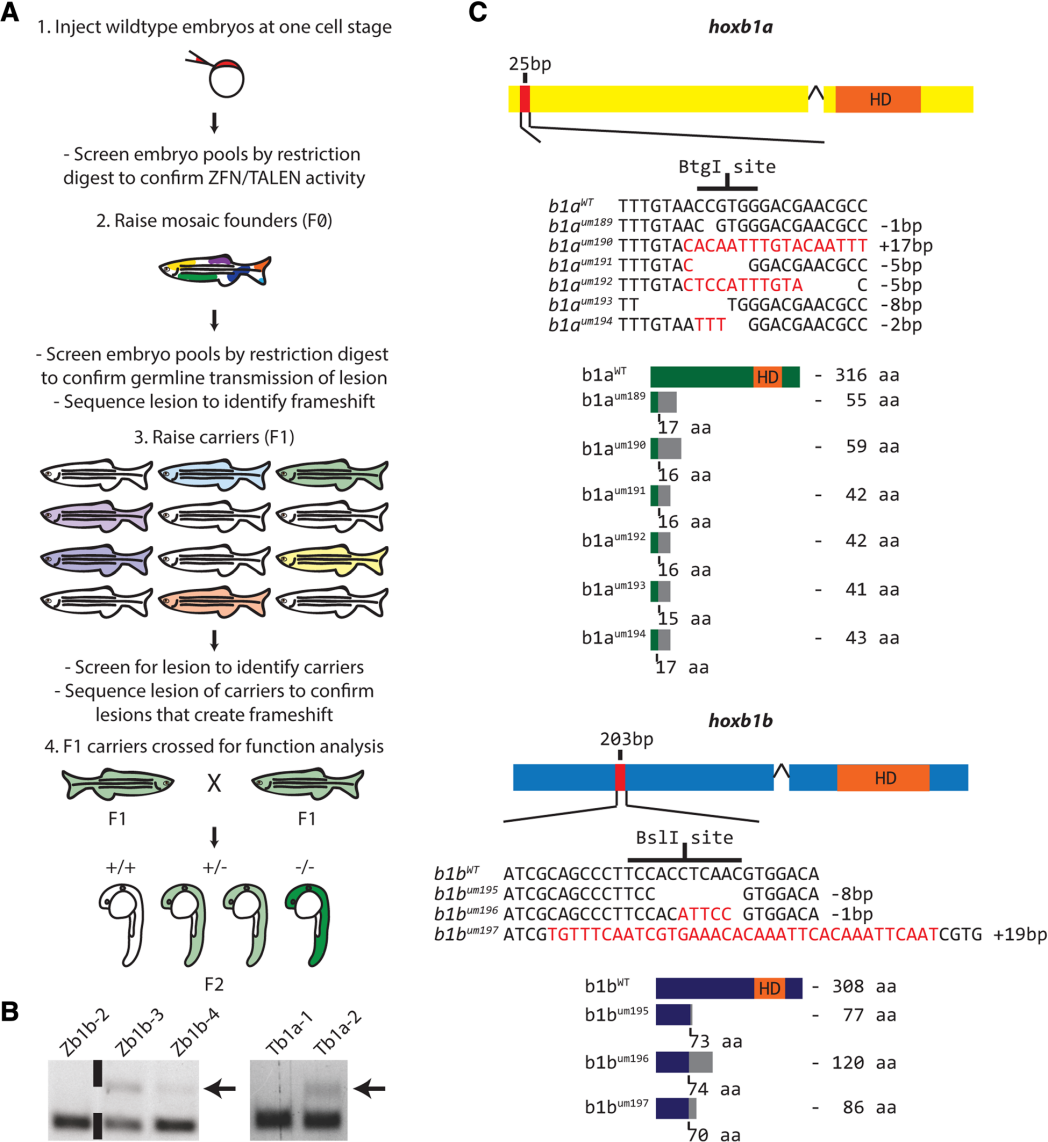
**Generation of *hoxb1a *and *hoxb1b *germ line mutants using ZFN and TALEN technologies. A**. Diagram outlining experimental strategy. Active ZFNs and TALENs were identified by their ability to disrupt the sequence of a diagnostic restriction site in genomic DNA. Embryos injected with active ZFNs and TALENs were then raised and screened for founders that transmit frameshift mutations via their germ line. **B**. Identification of active nucleases. Genomic DNA was prepared from pools of injected embryos and digested with a diagnostic restriction enzyme. The Zb1b-3, Zb1b-4 and Tb1a-2 injected pools contain undigested material (arrows), indicating that the diagnostic restriction site has been disrupted. **C**. Characterization of germ line transmitted *hoxb1a* and *hoxb1b* mutations. Six mutant *hoxb1a* (top) and three mutant *hoxb1b* (bottom) alleles were identified to cause frameshift mutations. Nucleotide and peptide sequences are indicated for each mutant allele. Gaps in the nucleotide sequence indicate deletions, while red nucleotides indicate insertions. Numbers to the right of each nucleotide sequence indicate the net size of insertions/deletions. For the peptide sequence, gray boxes indicate residues read out of frame prior to encountering a premature stop codon. Amino acid numbers below each peptide sequence indicate the residue affected by the frameshift mutation, while numbers to the right indicate the length of the mutant peptide. HD = homeodomain. Black wedge indicates site of single intron in each sequence.

In vitro transcribed mRNA encoding each ZFN pair was injected into early one-cell stage embryos and genomic DNA was prepared from pools of whole embryos collected 24 hours post fertilization (hpf; Figure 1A). ZFN activity was measured by amplifying the targeted region, followed by digestion to estimate the fraction of genome with a disruption of the diagnostic restriction site. Notably, the ZFNs are likely to act after the first several cell divisions (due to the rapid cell cycle of zebrafish embryos, as well as due to the need for the ZFN mRNA to be translated) and it is therefore expected that mutations will be induced in only a subset of cells – rendering the embryos mosaic. Embryos injected with Zb1b-3 and Zb1b-4, but not Zb1b-2 or Zb1b-1 (Figure 1B) revealed a partial loss of the diagnostic restriction site, suggesting that these ZFNs are active. Based on the intensity of the uncut band in the diagnostic digest, the Zb1b-3 ZFN may be more active than Zb1b-4. Since ZFNs from all three builds failed to induce mutations at the *hoxb1a* locus, we turned to TALENs as an alternative method to disrupt the *hoxb1a* gene. To increase the likelihood of success, we generated three different *hoxb1a* TALENs that differ slightly in the length of their target sequences (Table 1) using Golden Gate TALEN assembly [31]. As with ZFNs, TALENs were designed to target regions in the first exon of *hoxb1a* that include a diagnostic restriction site. Notably, the TALENs were directed to sites 450 bp (Tb1a-1) or 430 bp (Tb1a-2 and Tb1a-3) upstream from the region targeted by *hoxb1a* ZFNs. Using the same mRNA microinjection strategy as for the ZFNs, we found that TALEN Tb1a-2 introduced mutations – as evidenced by loss of the diagnostic restriction site – but that Tb1a-1 and Tb1a-3 did not (Figure 1B).

Having identified functional ZFNs and TALENs, we raised injected embryos to establish an adult F0 founder population. As noted, fish in this F0 population will be mosaic and each individual fish may carry more than one mutant allele for the same gene (Figure 1A). Genotyping of pooled offspring identified 20 *hoxb1a* (out of 24 tested) and 15 *hoxb1b* (out of 35 tested) F0 founders that transmit mutations via their germ lines, suggesting mutagenesis rates of 83% and 43% for *hoxb1a* and *hoxb1b*, respectively. Sequencing of mutant alleles from each F0 founder revealed that two of the 20 *hoxb1a* (A2 and A20) and three of the 15 *hoxb1b* (B2, B11 and B15) F0 founders carry mutations that introduce frame shifts (Table 2), while the remaining F0 founders transmitted mutations that disrupt the diagnostic restriction site, but that do not create frame shifts (two representative examples are shown in Additional file 1: Table S1). The five F0 fish that carry mutations causing frame shifts were outcrossed to wild type fish and the resulting offspring raised to generate the F1 generation. Genotyping of F1 fish allowed us to determine the transmission rate of mutations from mosaic F0 fish. We find that the *hoxb1b* F0 founders transmit their mutations at a frequency of ~40% (45% for B2, 43% for B11 and 41% for B15), while the *hoxb1a* F0 fish transmit their mutations at ~10% (14% for A2 and 9% for A20). Notably, we also find that the three *hoxb1b* founders each transmitted only one mutant allele, while the two *hoxb1a* founders transmitted multiple mutant alleles each (four alleles from A2 and two alleles from A20). Thus, we have generated six *hoxb1a* (*um189, um190, um191, um192, um193, um194)* and three *hoxb1b (um195, um196, um197)* mutant lines (Table 2) that were used for further phenotypic analysis.

**Table 2:** **Characteristics of *hoxb1a *** **and *hoxb1b *** **germ line mutant alleles**

**Founder**	**Transmission frequency**	**Sequence**^**a**^	**Type of mutation**^**b**^	**ID**^**c**^
***hoxb1a***		TTTGTAA**CCGTGG**GACGAACGCCTACT		
A2	14%	TTTGTAAC GTGGGACGAACGCCTACT	Deletion (−1 bp)	*um189*
		TTTGTA*CACAATTTGTACAATTT*GGACGAACGCCTACT	Insertion/deletion (+17 bp)	*um190*
		TTTGTA*CGGG* GGACGAACGCCTACT	Insertion/deletion (−5 bp)	*um191*
		TTTGTA*CTCCATTTGTA* CTACT	Insertion/deletion (−5 bp)	*um192*
A20	9%	TT TGGGACGAACGCCTACT	Deletion (−8 bp)	*um193*
		TTTGTAA**TTT**C GGACGAACGCCTACT	Insertion/deletion (−2 bp)	*um194*
***hoxb1b***		ATCGCAGCCCTTCCA**CCTCAACGTGG**ACATGGG		
B2	45%	ATCGCAGCCCTTCCACCTCAACGTGGACATGGG	Deletion (−8 bp)	*um195*
B11	43%	ATCGCAGCCCTTCCAC*ATTCC* GTGGACATGGG	Insertion/deletion (−1 bp)	*um196*
B15	41%	*TGTTTCAATCGTGAAACACAAATTCACAAATTCAAT*CGTGGACATGG	Insertion/deletion (+19 bp)	*um197*

^a^Sequence of mutant alleles. Deletions are shown as gaps and insertions are italicized. Nucleotides in bold indicate target site for diagnostic restriction enzyme in wild type sequence.

^b^Indicates whether mutation results from insertion, deletion or both. Numbers in parenthesis indicate net gain/loss of nucleotides in mutant sequence.

^c^Identifying designation for each mutant allele.

Closer analysis of the mutant sequences revealed that Zb1b-3 and Tb1a-2 generated both insertions and deletions (Figure 1C). In particular, the Zb1b-3 ZFN introduced deletions ranging from 1 bp to 8 bp, as well as a 19 bp insertion in the *hoxb1b* gene, while the Tb1a-2 TALEN introduced deletions ranging from 1 bp to 8 bp, as well as a 17 bp insertion, in the *hoxb1a* gene. We note that large insertions and deletions that interfere with the PCR reaction (e.g. by deleting a primer site) would not be detected by our experiments, suggesting that the sizes observed here may be somewhat biased to smaller deletions and insertions. Conceptual translation of each mutant allele confirmed a shift in the reading frame (Figure 1 and Additional file 2: Figure S1). As a result, *hoxb1a* mutant alleles go out of frame starting with residue 15 (*um193*), residue 16 (*um190*, *um191* and *um192*) or residue 17 (*um189* and *um194*) and *hoxb1b* mutants starting with residue 71 (*um197*), residue 74 (*um195*) or residue 75 (*um196*). While the out of frame sequences code for varying numbers of missense residues, all terminate in a premature stop codon and none of the mutant alleles is predicted to encode a homeodomain.

Lastly, we raised embryos from *hoxb1a*^*+/um191*^ x *hoxb1a*^*+/um192*^ and *hoxb1b*^*+/um197*^ x *hoxb1b*^*+/um197*^ crosses to adulthood and genotyped them. We find that *hoxb1a*^*um191/um192*^ animals do not survive to adulthood (0/23 genotyped adults were *hoxb1a*^*um191/um192*^), while *hoxb1b*^*um197/um197*^ embryos do – although their viability may be somewhat reduced (13/84 genotyped adults were *hoxb1b*^*um197/um197*^). We note that this is in contrast to the situation in the mouse, where both *Hoxa1*^*−/−*^ and *Hoxb1*^*−/−*^ pups die shortly after birth [20,26].

### *hoxb1b* is required for zebrafish hindbrain segmentation

Formation of the vertebrate hindbrain requires segmentation of the neural tube into rhombomere domains, as well as the specification of distinct cell fates and the differentiation of characteristic types of neurons in each rhombomere. Paralog group 1 (PG1) *hox* genes, such as *hoxb1a* and *hoxb1b*, are among the earliest genes expressed in the hindbrain primordium and *hox* function has been implicated in multiple aspects of hindbrain development (reviewed in [16]). We therefore made use of the *hoxb1a* and *hoxb1b* mutant lines to examine the role of these genes in development of the zebrafish hindbrain.

**Figure 2:**
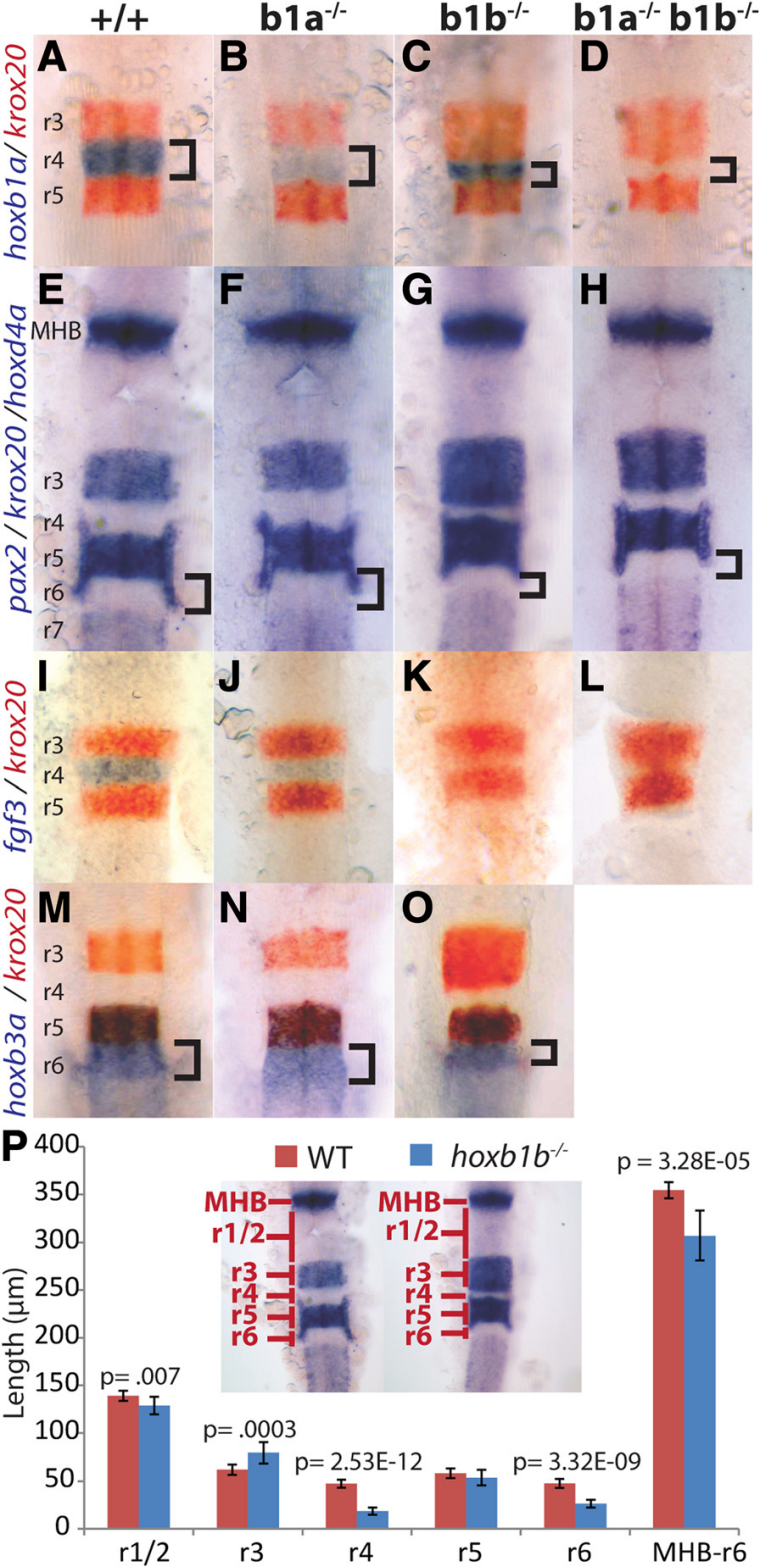
**Zebrafish *hoxb1b *is required for hindbrain segmentation. A-O**. Wild type **(A, E, I, M)**, *hoxb1a*^*−/−*^**(B, F, J, N)**, *hoxb1b*^*−/−*^**(C, G, K, O)** or doubly *hoxb1a*^*−/−*^;*hoxb1b*^*−/−*^ embryos **(D, H, L)** were assayed by in situ hybridization for expression of *hoxb1a* in r4 (blue stain in **A-D**), *krox20* in r3/r5 (red stain in **A-D**, **I-O** and blue stain in panels **E-H**), *pax2* at the midbrain-hindbrain boundary (blue stain in **E-H**), *fgf3* in r4 (blue stain in **I-L**), *hoxd4a* in r7 (blue stain in **E-H**) and *hoxb3a* in r5/r6 (blue stain in **M-O**). **P**. Quantification of segmentation defects in *hoxb1b*^*−/−*^ embryos. Rhombomere lengths were measured as indicated in the inset. MHB-r6 measures the full distance from the anterior limit of the MHB to the posterior limit of r6. p-values were computed using Students’ *t*-test and error bars represent standard error. N = 10 embryos. All embryos are flat mounted in dorsal view with anterior to the top. A-H and M-O are at 22hpf, while I-L are at 14hpf. r = rhombomere; MHB = midbrain/hindbrain boundary.

We first examined the expression of several rhombomere-restricted genes – *pax2* (expressed at the midbrain-hindbrain boundary; MHB), *krox20* (expressed in r3 and r5), *hoxb1a* (expressed in r4), *hoxb3a* (expressed in r5 and r6) and *hoxd4a* (expressed in r7 and r8). For this purpose, heterozygous *hoxb1b*^*+/um197*^ F1 fish were in-crossed and the resulting embryos were assayed at 22hpf by in situ hybridization followed by genotyping. We find that homozygous *hoxb1b*^*um197/um197*^ mutant embryos express *krox20* in r3 and r5, as well as *hoxb1a* in r4 (Figure 2C). However, the size of r3 is increased and the size of r4 is decreased in *hoxb1b* mutants relative to wild type (or heterozygous) embryos (Figure 2A; Additional file 3: Table S2). To address the possibility that the Zb1b-3 ZFN might have introduced off-target mutations in the *hoxb1b*^*um197*^ line that could contribute to this phenotype, we also examined in-crosses of the *hoxb1b*^*um196*^ and *hoxb1b*^*um195*^ lines, as well as pair-wise inter-crosses among all three lines. We find that mutant embryos derived from all such crosses exhibit the same phenotype and that this phenotype segregates with the *hoxb1b* mutation (Additional file 3: Table S2 and Additional file 4: Figure S2), suggesting that it is due to disruption of the *hoxb1b* gene. Furthermore, the PROGNOS on-line tool [37] revealed five exonic sites in the top fifty potential off-target sites for Zb1b-3, but neither of these sites resides on the same chromosome as the *hoxb1b* gene, and they would therefore segregate independently of the *hoxb1b* mutation in our crosses. Further analysis of *hoxb1b*^*um197/um197*^ mutant embryos revealed expression of *pax2*, *hoxb3a* and *hoxd4a* in the expected domains (Figure 2G, O). In addition to the enlargement of r3 and the reduction of r4 noted above, this analysis also revealed an apparent reduction of r6 – as evidenced by a smaller gap between r5 *krox20* staining and r7 *hoxd4a* staining (brackets in Figure 2E, G), as well as by a reduction in the size of the *hoxb3a* expression domain (brackets in Figure 2M, O) in mutant embryos relative to wild type embryos.

We next quantified the changes in rhombomere size by direct measurements (Figure 2P). We find that r3 is significantly enlarged (79.4 μm in *hoxb1b* mutants versus 62.0 μm in wild type; p = 0.0003) and r4 significantly reduced (18.5 μm in mutant versus 47.4 μm in wild type; p = 2.53E-12) in mutant embryos. Notably, we cannot distinguish whether this effect is due to some cells switching from an r4 to an r3 fate, or if r3 cells have a growth advantage in the absence of *hoxb1b* function. We also find that r6 (26.5 μm in mutant versus 47.6 μm in wild type; p = 3.32E-09) and r1/r2 (129 μm in mutant versus 139 μm in wild type; p = 0.007) are somewhat reduced, but r5 is unaffected, in *hoxb1b* mutants. Accordingly, measuring the length of the entire hindbrain reveals it to be significantly shorter in *hoxb1b* mutant embryos (306 μm in mutant versus 354 μm in wild type; p = 3.28E-05), presumably as a result of the reduced length of several rhombomeres.

Analysis of *hoxb1a* mutant embryos (derived from an inter-cross of *hoxb1a*^*um191/+*^ and *hoxb1a*^*um192/+*^ carriers) revealed normal expression of *krox20* in r3 and r5 of the hindbrain (Figure 2B, F, J, N). Furthermore, the size of the r4 domain is normal, but expression of *hoxb1a* is markedly reduced in r4 of *hoxb1a* mutants (Figure 2B; Additional file 3: Table S2). Examination of embryos derived from inter-crosses of the *hoxb1a*^*um189*^*, hoxb1a*^*um190*^, *hoxb1a*^*um193*^, and *hoxb1a*^*um194*^ alleles confirmed this phenotype and suggest that it results from mutation of *hoxb1a* rather than from off-target mutations introduced by the Tb1a-2 TALEN (Additional file 3: Table S2 and Additional file 5: Figure S3). Accordingly, the PROGNOS tool did not reveal any exonic sites among the top fifty potential off-target sites for the Tb1a-2 TALEN. Since *hoxb1a* regulates its own expression [38], the loss of *hoxb1a* transcript in *hoxb1a* mutants may be due to the loss of Hoxb1a protein, or, alternatively, to reduced stability of the mutant *hoxb1a* transcript. Accordingly, expression of *fgf3*, another r4-restricted gene dependent on *hoxb1a* for expression, is also markedly reduced in r4 of *hoxb1a* mutants at 14hpf (Figure 2J). Further analyses demonstrated normal expression of *pax2, hoxd4a* and *hoxb3a* (Figure 2F, N) and also revealed normal size of rhombomeres in *hoxb1a* mutant embryos. We conclude that *hoxb1b* is required for formation of appropriately sized rhombomere segments in the caudal hindbrain. In contrast, *hoxb1a* is essential in regulating expression of r4-restricted genes, indicating a role for *hoxb1a* in r4 specification.

### *hoxb1a* is required for formation of r4-derived neurons

**Figure 3:**
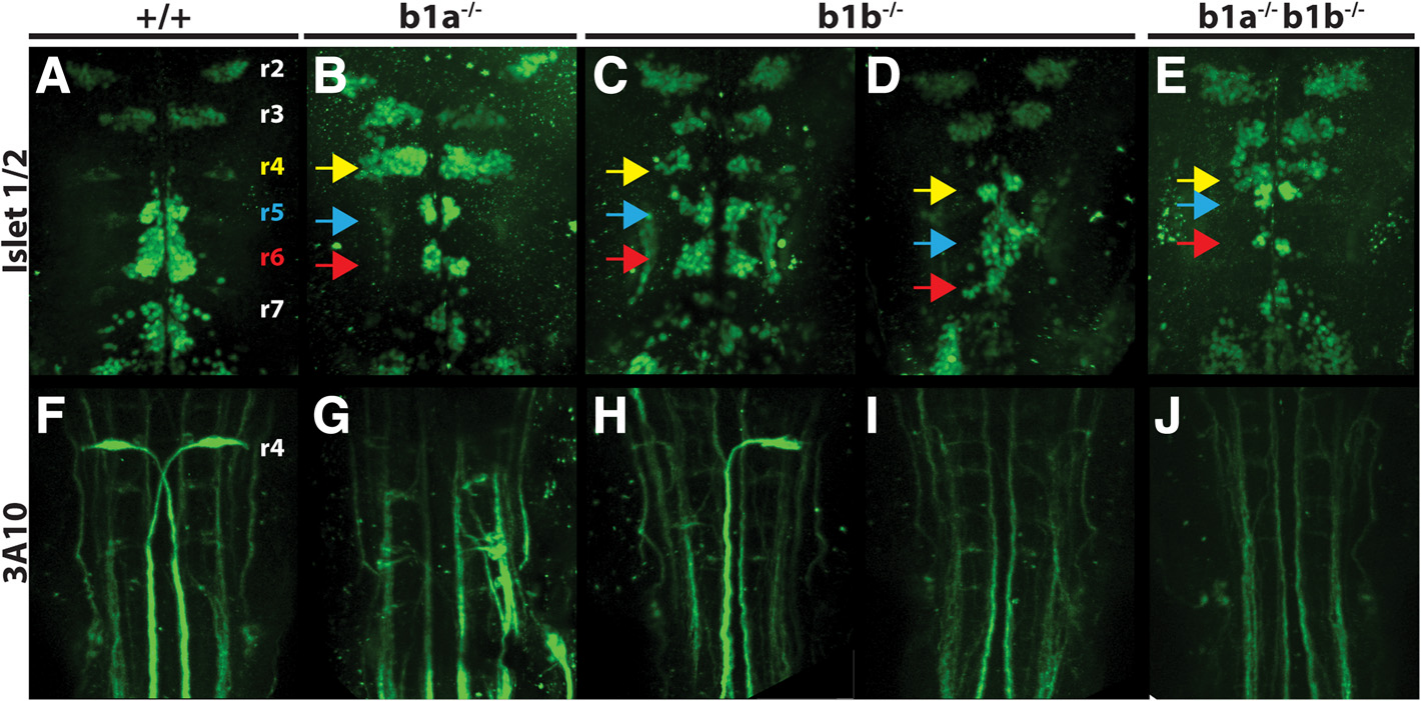
***hoxb1a *and *hoxb1b *are required for neuronal differentiation in the hindbrain.** 48hpf wild type **(A, F)**, *hoxb1a*^*−/−*^**(B, G)**, *hoxb1b*^*−/−*^**(C, D, H, I)** or doubly *hoxb1a*^*−/−*^;*hoxb1b*^*−/−*^ embryos **(E, J)** were assayed by immunostaining for the differentiation of branchiomotor neurons (islet1/2 staining in **A-E**) and Mauthner neurons (3A10 staining in **F-J**). Colored arrowheads indicate r4 (yellow), r5 (blue) and r6 (red). All embryos are flat mounted in dorsal view with anterior to the top.

A key event in hindbrain development is the differentiation of unique complements of neurons in each rhombomere. In particular, motor neurons of the V^th^ (trigeminal) cranial nerve differentiate in r2 and r3, motor neurons of the VI^th^ (abducens) cranial nerve form in r5 and r6 and motor neurons of the X^th^ (vagal) cranial nerve form in the caudal most region of the hindbrain. In addition, motor neurons of the VII^th^ (facial) cranial nerve form in r4, but subsequently migrate to r6 and r7 in zebrafish. In order to determine if neuronal differentiation is affected in *hoxb1a* and *hoxb1b* mutants, we analyzed cranial motor neuron formation. Wild type embryos displayed the expected stereotypical arrangement of cranial motor neurons at 48hpf (Figure 3A). Notably, this includes an almost complete lack of facial motor neurons (FMNs) in r4 as a result of these neurons having migrated caudally by this stage. In contrast, all *hoxb1a*^*um191/um192*^ mutant embryos revealed a large number of motor neurons in r4 and reduced numbers in r6 and r7 (Figure 3B). We interpret this to mean that FMNs fail to migrate out of r4 and that the residual motor neurons in r5/r6 primarily represent abducens neurons. FMNs also remain in r4 of all *hoxb1b* mutant embryos, but the phenotype is more variable than what is observed in *hoxb1a* mutants. Approximately half of the *hoxb1b* mutants reveal significant retention of FMNs in r4 (Figure 3C), while the other half shows more extensive FMN migration (Figure 3D). In addition to impaired FMN migration from r4, the number of FMNs appears lower in *hoxb1b* mutants and these are less well organized, with cells being less tightly grouped in r5-r7 and numerous cells found located outside the main clusters.

Similar to the cranial motor neurons, reticulospinal neurons also display rhombomere-specific differentiation in zebrafish. Specifically, bilaterally arranged Mauthner neurons form in r4 and project their axons across the midline down into the spinal cord at 36hpf (Figure 3F). Notably, differentiated Mauthner neurons are absent in *hoxb1a* mutant embryos (Figure 3G). As observed for the cranial motor neurons, *hoxb1b* mutants show a variable phenotype such that approximately half of the embryos retain one Mauthner neuron, while the other half fails to form both Mauthner neurons (Figure 3H, I). We conclude that *hoxb1a* function is absolutely required for FMN migration and Mauthner neuron formation, but that *hoxb1b* is only partially required for these processes.

### *hoxb1a* and *hoxb1b* have separate functions in zebrafish hindbrain development

Since *hoxb1a* and *hoxb1b* are both required for normal r4 formation, we examined their functional relationship by analyzing *hoxb1a/hoxb1b* double mutant embryos (generated from a cross between *hoxb1a*^*+/um193*^*; hoxb1b*^*+/um197*^ and *hoxb1a*^*+/um194*^*; hoxb1b*^*+/um197*^ double heterozygotes). Using in situ hybridization, we find that double mutant embryos have hindbrain segmentation defects with an expansion of r3 and a reduction of r4 and r6 (Figure 2D, H; Additional file 3: Table S2). These changes in rhombomere size are indistinguishable from those observed in *hoxb1b* mutant embryos (compare Figure 2G to H), further demonstrating that *hoxb1a* does not play a role in zebrafish hindbrain segmentation. Furthermore, double mutant embryos lack *hoxb1a* expression, while single mutants show a reduction either in the level (*hoxb1a* mutants) or domain size (*hoxb1b* mutants) of *hoxb1a* expression (compare Figures 2B-D), indicating that both genes may play a role in *hoxb1a* transcription.

Next we examined neuronal differentiation in double mutant embryos. We find that FMNs form in r4 of double mutants, but do not migrate caudally (Figure 3E), similar to the phenotype of *hoxb1a* mutants (Figure 3B). We also note that the population of FMNs is smaller in double mutants (Figure 3E), similar to the phenotype of *hoxb1b* mutants (Figure 3C, D). Lastly, double mutants completely lack Mauthner neuron formation in r4 (Figure 3J), identical to the *hoxb1a* mutant phenotype. These findings are consistent with *hoxb1b* being required for the size of r4 (and therefore the number of neurons formed in r4), while *hoxb1a* is required for the migration of FMNs and the differentiation of Mauthner neurons. Notably, the abducens neuron population in r6 may be slightly smaller in double mutants, consistent with r6 being smaller in the absence of *hoxb1b*, but this effect cannot be seen in *hoxb1b* single mutants due to the residual migrating FMNs. We conclude that *hoxb1a* and *hoxb1b* have different functions in hindbrain development.

### *hoxb1b* and retinoic acid independently activate *hoxb1a* expression

Although the size of r4 is reduced in *hoxb1b* mutant embryos, we note that *hoxb1a* expression persists. The expression of *hoxb1a* in the absence of *hoxb1b* is somewhat surprising since previous work suggested that early neural expression of *hoxb1a* depends on a Hoxb1b-regulated enhancer [29]. Since retinoic acid (RA) is known to activate *hox* gene transcription in many settings [13,25,28,39-43], we next investigated whether RA signaling activates *hoxb1a* transcription in the absence of *hoxb1b*.

**Figure 4:**
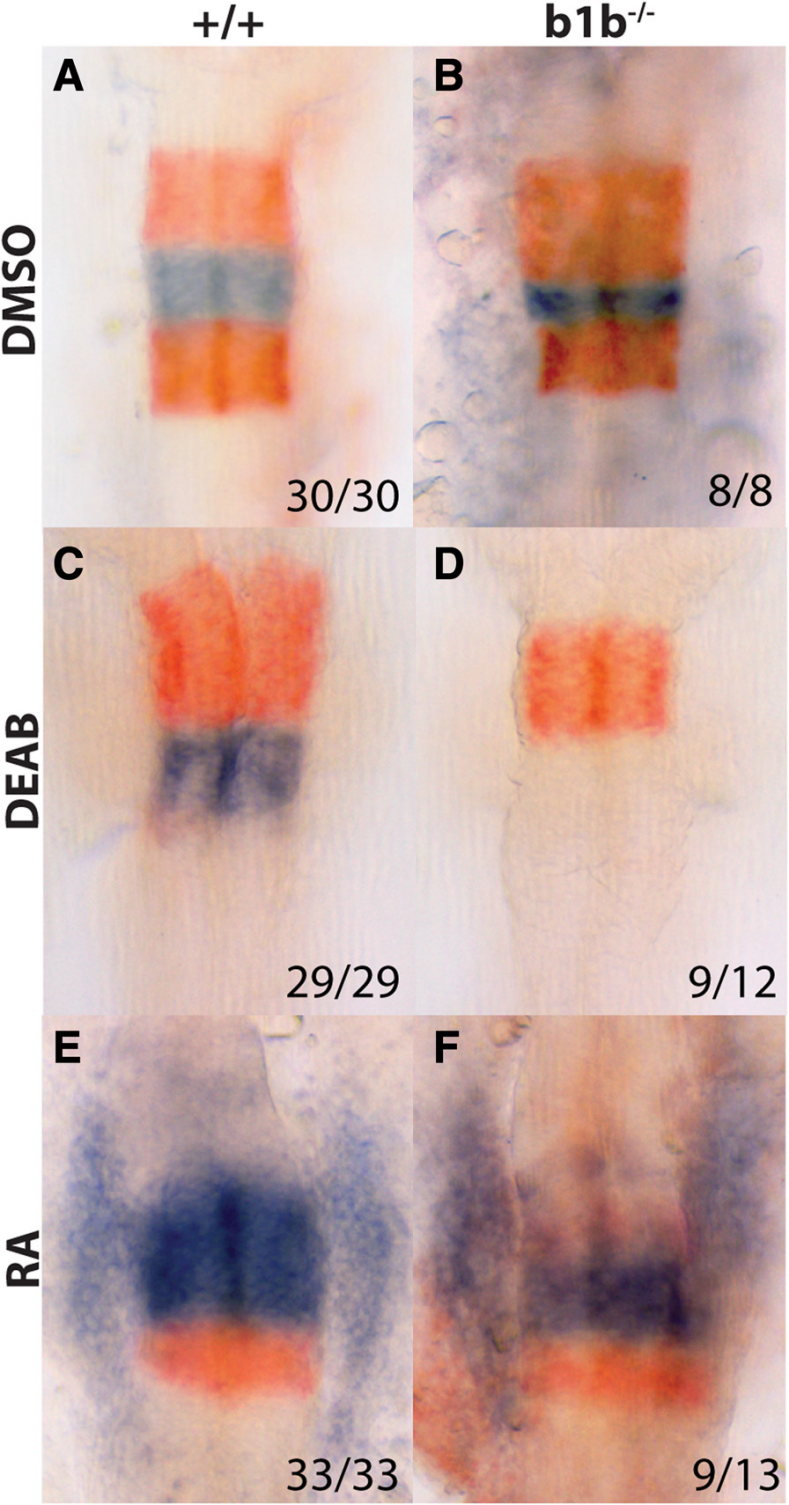
**Retinoic acid and *hoxb1b *act independently to activate *hoxb1a *transcription.** 19hpf wild type **(A, C, E)** or *hoxb1b*^*−/−*^**(B, D, F)** embryos were treated with DMSO (control; **A, B**), 10uM DEAB **(C, D)** or 100 nM RA **(E, F)** and assayed by in situ hybridization for expression of *hoxb1a* in r4 (blue stain in **A-F**) and *krox20* in r3/r5 (red stain in **A-F**). All embryos are flat mounted in dorsal view with anterior to the top.

To this end, embryos were treated with 10uM diethylaminobenzaldehyde (DEAB; a small molecule inhibitor of the RALDH enzyme involved in RA synthesis), or with 100nM exogenous retinoic acid, from 1hpf to 19hpf (Figure 4). We find that, in wild type embryos, 10uM DEAB blocks *krox20* expression in r5, though not in r3 (that instead appears to be expanded), but does not affect *hoxb1a* expression in r4 (Figure 4C). Treatment of wild type embryos with RA produces a distinct phenotype such that *hoxb1a* expression is expanded, while *krox20* expression is lost in r3 and reduced in r5 (Figure 4E). These results indicate that RA supports *krox20* expression in r5 while it inhibits it in r3, consistent with previous reports that RA promotes posterior fates [44,45]. Furthermore, while *hoxb1a* expression in wild type embryos is enhanced by exogenous RA, it is not lost upon treatment with 10uM DEAB.

In *hoxb1b*^*um197/um197*^ mutants, DEAB treatment blocks r5 *krox20* expression – similar to the effect in wild type embryos – while r3 size is reduced somewhat (Figure 4D). However, while DEAB treatment has no effect on *hoxb1a* expression in wild type embryos, it blocks expression in *hoxb1b* mutants (Figure 4D). In contrast, RA treatment has the same effect in *hoxb1b* mutants as in wild type embryos, in that *krox20* expression is lost in r3 and reduced in r5, while *hoxb1a* expression is expanded (Figure 4F). Taken together, our results reveal that *hoxb1a* expression is abolished upon simultaneous removal of *hoxb1b* and RA, but not when either factor is removed by itself, suggesting that RA and *hoxb1b* independently activate *hoxb1a* expression in zebrafish r4.

### *hoxb1b* affects nucleosome positioning at the promoter of *hoxb1a*

**Figure 5:**
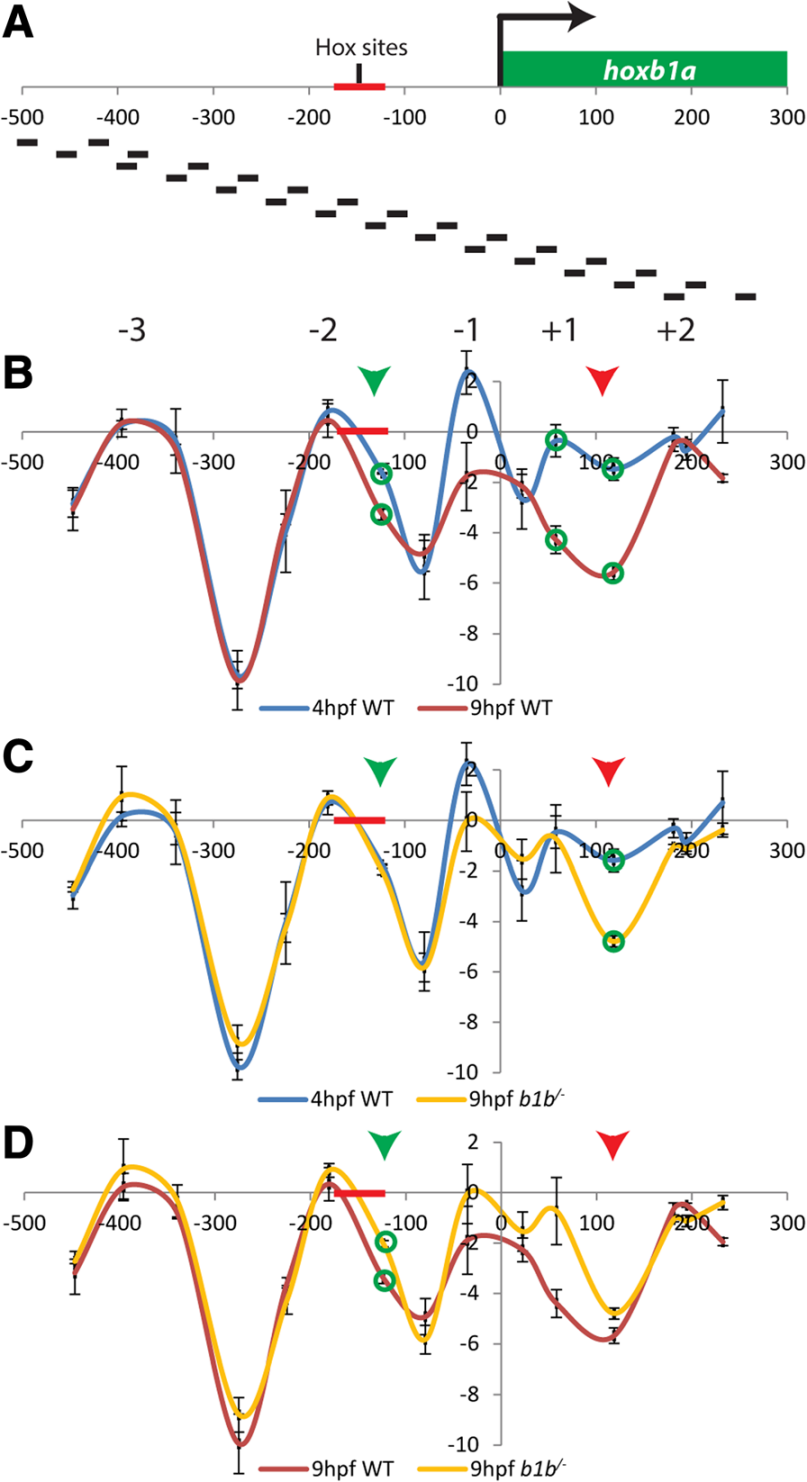
**Hoxb1b affects nucleosome organization at the *hoxb1a *promoter. A**. Diagram illustrating location of primers used for nucleosome scanning across the *hoxb1a* locus. **B-D**. Overlay of nucleosome profiles at the *hoxb1a* promoter for 4hpf versus 9hpf wild type embryos **(B)**, 4hpf wild type versus 9hpf *hoxb1b*^*−/−*^ embryos **(C)** and 9hpf wild type versus 9hpf *hoxb1b*^*−/−*^ embryos **(D)**. The −3, −2, −1, +1 and +2 nucleosomes are labeled in **(B)**. Green circles indicate locations where changes in nucleosome organization are statistically significant (p < 0.05) while arrowheads point to Site 1 (green arrowhead) and Site 2 (red arrowhead) discussed in the text. Error bars represent standard error of three biological replicates. Red region on horizontal axis indicates approximate location of Pbx/Meis/Hox binding sites.

We recently found that nucleosome positioning at the promoter regions of zebrafish *hox* genes is a progressive process that occurs over several stages of embryogenesis independent of RA signaling [46]. Since Hoxb1b regulates *hoxb1a* expression, at least in part, we wanted to test if Hoxb1b plays a role in nucleosome positioning at the *hoxb1a* promoter. We took advantage of the fact that *hoxb1b*^*um197/um197*^ mutant fish are viable and mapped nucleosomes at the *hoxb1a* promoter of WT and *hoxb1b* mutant embryos using a modified nucleosome scanning approach (Figure 5A; [47]).

We detect three well-positioned nucleosomes at approximately 360 bp, 180 bp and 35 bp upstream of the transcription start site (TSS; −3, −2, and −1 nucleosomes; Figure 5B), as well as two less well-defined nucleosomes at 50 bp and 200 bp downstream of the TSS (+1 and +2 nucleosomes) at 4hpf – a stage when Hoxb1b protein is not yet expressed and cannot yet be detected at the *hoxb1a* promoter [48]. The nucleosome profile at 9hpf – when Hoxb1b is present at the *hoxb1a* promoter [48] – is similar to that at 4hpf, but there are some notable differences (Figure 5B). In particular, there is reduced nucleosome density surrounding the TSS from approximately position -200 bp to position +200 bp, indicating that changes in nucleosome density occur at the *hoxb1a* promoter at developmental time points when Hoxb1b is bound at the promoter.

To determine if the change in nucleosome arrangement between 4hpf and 9hpf is driven by Hoxb1b, we compared the nucleosome profile of *hoxb1b* mutant embryos at 9hpf to that of wild type embryos at 4hpf (Figure 5C). If Hoxb1b is solely responsible for promoting changes in nucleosome arrangement, the two profiles should be similar. Indeed, the 9hpf *hoxb1b* mutant and 4hpf wild type profiles are more similar to each other than are the wild type 9hpf and wild type 4hpf profiles, with the exception of a region centered at approximately +120 bp downstream of the TSS (Site 2; red arrow). This finding suggests that Hoxb1b is responsible for affecting nucleosome organization at the TSS and immediately upstream, but not further downstream at Site 2. Such a scenario is consistent with the fact that Hoxb1b is known to bind regulatory elements located 250-150 bp upstream of the *hoxb1a* TSS.

Accordingly, when we compare the nucleosome profile of 9hpf *hoxb1b* mutants to that of 9hpf wild type embryos, we find that the region around +120 bp (Site 2) is relatively unaffected, again consistent with nucleosomes in this region being organized independently of Hoxb1b (Figure 5D). As expected, there remains a significant difference upstream of the TSS (Site 1, green arrow) between 9hpf wild type and 9hpf mutant embryos, confirming that nucleosome occupancy in this region is likely dependent on Hoxb1b binding. Together, these data identify two regions where nucleosome organization is affected at the *hoxb1a* promoter. The first, ~120 bp upstream of the *hoxb1a* TSS (Site 1), near the known Hoxb1b binding site appears to be directly affected by Hoxb1b binding, while the second, ~120 bp downstream of the TSS (Site 2), appears to be independent of Hoxb1b binding.

## Discussion

The phenotypes of mouse *Hoxa1* and *Hoxb1* germ line mutants, as well as the phenotypes of anti-sense mediated knock-down of zebrafish *hoxb1b* and *hoxb1a* have been characterized [20-24,26-29,43]. Differences between the zebrafish and mouse phenotypes suggest that these genes may play different roles in different species, or, possibly, that zebrafish phenotypes induced by anti-sense morpholinos (MOs) may not represent complete loss of function. To clarify these differences we created targeted germ line mutations for zebrafish *hoxb1b* and *hoxb1a* with ZFN and TALEN systems, respectively. Our findings indicate that *Hoxa1* and *hoxb1b* share roles in hindbrain segmentation and that *Hoxb1* and *hoxb1a* have similar roles in facial motor neuron migration. Comparing the phenotypes of our germ line mutants to those of MO loss-of-function suggests that the MO phenotypes do not represent complete loss of function in all respects, although we cannot completely rule out the possibility that differences in genetic backgrounds may also affect the observed differences. Our experiments also reveal that *hoxb1b* and *hoxb1a* have species-specific functions. In addition, we report that *hoxb1a* transcription is independently regulated by both RA and Hoxb1b. Lastly, we demonstrate that Hoxb1b mediates its effect on *hoxb1a* expression, at least in part, by affecting nucleosome positioning at the *hoxb1a* promoter.

### *Hoxa1* and *hoxb1b* have universal as well as species-specific roles in hindbrain segmentation

Comparison of the previously reported hindbrain phenotypes of mouse *Hoxa1* (produced by targeted germ line disruption) and zebrafish *hoxb1b* (produced by anti-sense MO) loss-of-function studies reveal several similarities. In particular, r3 is expanded while r4 and r5 are reduced in both species [20-22,24,28,29]. However, there are differences in the segmentation defects observed. In particular, the r5 segmentation defect in mouse *Hoxa1*^*−/−*^ mutants is more severe, with some embryos losing r5 entirely [22], while *hoxb1bMO* zebrafish embryos display a reduction, but not a loss, of r5 [29]. In *hoxb1bMO* embryos r6 is also reduced, a phenotype not observed in *Hoxa1*^*−/−*^ mice. The *hoxb1b* germ line mutants presented here have an expanded r3 and a reduced r4, similar to mouse *Hoxa1* mutants and *hoxb1bMO* embryos, indicating that *Hoxa1* and *hoxb1b* share universal roles in the formation of these rhombomeres. However, germ line disruption of *hoxb1b* produces a more severe phenotype than MO injection – e.g. Mauthner neurons are affected in the germ line mutant, but not by Hoxb1b MO – suggesting that the Hoxb1b MO does not completely block Hoxb1b function. Lastly, we observe a fully formed r5 and a reduced r6 in *hoxb1b* germ line mutants, distinct from mouse *Hoxa1* mutants, suggesting that the loss of r5 in *Hoxa1*^−/−^ mice is species specific and that *hoxb1b* has a species-specific role in r6 of zebrafish. We note that a zebrafish *hoxb1b* TILLING mutant was recently published [49]. While the effects on anteroposterior patterning were not characterized in detail for this mutant, Mauthner neuron formation was affected and it appears that r4 may be reduced in size – consistent with the phenotype of our *hoxb1b* ZFN alleles.

### *Hoxb1* and *hoxb1a* are required for r4 specification

Previous reports indicate that mouse *Hoxb1* and zebrafish *hoxb1a* share functions important for the migration of facial motor neurons (FMNs) from r4 during vertebrate hindbrain development [26,27,29] and we confirm this function in the *hoxb1a* germ line mutants reported here. However, it has not been clear if *hoxb1a* is absolutely required for r4 specification in zebrafish. In particular, r4 specification can be tested further in zebrafish by assaying the formation of Mauthner neurons. In previous work, embryos injected with *hoxb1aMO* were found to have normal Mauthner neurons (McClintock et al., 2002) – suggesting either that *hoxb1a* is not absolutely required in r4, or that the MO-injections do not produce a complete null phenotype. Strikingly, we find that Mauthner neurons fail to form in *hoxb1a* germ line mutant embryos, indicating that the *hoxb1aMO* does not completely eliminate *hoxb1a* activity. It is not clear what step in Mauthner neuron differentiation is affected by loss of *hoxb1a* function, but the absence of Mauthner neurons was accompanied by the appearance of smaller neurons at the equivalent position in embryos co-injected with *hoxb1aMO* and *hoxb1bMO*[29], potentially suggesting a fate change or a failure to differentiate. Since our *hoxb1a* germ line mutant embryos also lack expression of the r4-specific *fgf3* gene, we conclude that *hoxb1a* is required for r4-specific gene expression and neuronal differentiation in zebrafish.

### Regulation of *hoxb1a* transcription by RA and Hoxb1b

Initiation of *Hoxb1*/*hoxb1a* transcription can be mediated by Hoxa1/Hoxb1b binding with Pbx and Meis/Prep cofactors at a Pbx/Prep/Hox responsive element (r4-regulatory element) located upstream of the *Hoxb1/hoxb1a* TSS [38,50-56]. In addition, expression of mouse *Hoxb1* depends on a RA response element (RARE) located 3’ of the gene [43,57], but it is unclear if such an element is functional at the zebrafish *hoxb1a* locus [29,58], suggesting that *hoxb1a* might instead be highly dependent on *hoxb1b* for its expression in zebrafish. While we find that the *hoxb1a* expression domain is smaller in *hoxb1b* mutants, the level of expression does not appear noticeably affected, demonstrating that *hoxb1a* expression can be activated independently of Hoxb1b in zebrafish. Treatment with DEAB revealed that the *hoxb1a* expression observed in *hoxb1b* mutants requires RA signaling. Hence, our data suggest that Hoxb1b and RA can act independently to activate *hoxb1a* expression and that they are the predominant factors involved in this process – since simultaneous removal of both factors abolishes *hoxb1a* transcription. However, it remains possible that some aspects of *hoxb1a* expression require cooperation of RA and *hoxb1b* – e.g. RA induces *hoxb1a* more broadly in wild type than in *hoxb1b* mutant embryos. Since a functional RARE has not been identified at the *hoxb1a* locus, it is not clear whether RA acts directly, or if it acts indirectly via an intermediate factor.

As discussed, Hoxb1b regulates *hoxb1a* transcription by forming a complex with Pbx and Prep/Meis factors at the r4 regulatory element upstream of the *hoxb1a* TSS [48,54]. We recently demonstrated that nucleosome positioning around the promoters of *hox* genes is influenced by DNA-binding factors during early embryogenesis, but that RA signaling does not appear to affect this process [46]. We therefore took advantage of the fact that homozygous *hoxb1b*^*um197/um197*^ mutant fish are viable to test if Hoxb1b affects nucleosome organization at the *hoxb1a* promoter. We find high nucleosome density across the *hoxb1a* TSS at 4hpf (when Hoxb1b is not yet bound to the promoter and *hoxb1a* is not yet expressed), but this density is reduced in a region extending from +200 bp to -200 bp in wild type embryos at 9hpf (when Hoxb1b is bound to the promoter and *hoxb1a* is expressed). Strikingly, the affected region upstream of the *hoxb1a* TSS is near the r4 regulatory element (that is known to bind Hoxb1b) and nucleosome density in this region is elevated in *hoxb1b* mutants at 9hpf, suggesting that Hoxb1b is involved in depleting nucleosomes in this region coincident with onset of *hoxb1a* transcription. In contrast, a region downstream of the TSS undergoes a reduction in nucleosome density by 9hpf in both *hoxb1b* mutant and wild type embryos, suggesting that nucleosome changes in this region are independent of *hoxb1b* and must depend on other factors. Taken together, our findings indicate that *hoxb1a* expression is regulated both by Hoxb1b acting, at least in part, to affect nucleosome organization and by RA acting via an unknown pathway.

## Conclusions

We generated several novel germ line mutants for zebrafish *hoxb1a* and *hoxb1b.* Our analyses indicate that mouse *Hoxb1* and zebrafish *hoxb1a* have comparable functions, suggesting a conserved role for these genes. In contrast, while mouse *Hoxa1* and zebrafish *hoxb1b* share functions in the formation of r3 and r4, they differ with regards to r5 and r6, where *Hoxa1* appears to control formation of r5, but not r6, in the mouse, whereas *hoxb1b* regulates formation of r6, but not r5, in zebrafish. Lastly, our data reveal independent regulation of *hoxb1a* expression by retinoic acid and Hoxb1b in zebrafish.

## Methods

### Fish care

Ekkwill (EK) embryos were collected through natural matings and staged using morphological criteria as defined previously [59].

### Generation of zinc finger and Tale nucleases

Zinc finger nucleases (ZFNs) in build 1 and 2 (Table 1) were constructed from a single finger modular archive [35]. ZFNs for build 3 were designed from a library containing optimized two-finger modules for improved DNA recognition [36]. Nuclease assemblies for all three builds were completed using previously published protocols [35,36]. TALENs were constructed using the Golden Gate TALEN assembly kit (addgene: TALEN Kit #1000000024) following previously published protocols [31,60]. pCS2 plasmids containing completed ZFNs and TALENs were linearized and in vitro transcription was performed with the T7 mMachine ultra kit (Ambion: AMB1345). 50-100 pg of mRNA encoding ZFNs or TALENs was then injected into wild type embryos at the one cell stage.

### Identification of germ line mutations

Genomic DNA (gDNA) was prepared from a pool of 50 injected embryos at 24hpf. A fragment overlapping the nuclease target site (a 200 bp fragment carrying a BtgI restriction site from exon one of *hoxb1a* and a 300 bp fragment carrying a BslI restriction site from exon one of *hoxb1b*; see Additional file 6: Table S3 for primer sequences) was amplified and digested to determine if the diagnostic restriction site had been disrupted, indicating an active nuclease. Embryos injected with active TALENs and ZFNs were raised to adulthood. These mosaic founders (F0) were outcrossed to wild type fish and pools of 50 embryos were genotyped as above to identify germ line transmission. An F1 generation was raised from each germ line positive founder and F1 carriers were identified by genotyping of gDNA isolated from fin clips [61]. Individual F1 carriers were then sequenced to determine if a frameshift had occurred. Carriers with frameshift mutations were used for phenotypic analysis.

### Genotyping

Sequencing of F1 fish carrying frame shift mutations revealed the introduction of a BtgI restriction site in the *hoxb1b*^*um195*^ and *hoxb1b*^*um196*^ lines that was used for subsequent genotyping. Further, *hoxb1b*^*um197*^ contains an insertion that was used for subsequent genotyping by employing an insertion-specific primer. See Additional file 6: Table S3 for primer information.

### In situ probes and antibody labeling

In situ protocols were as previous published [62]. Embryos were fixed in 4% paraformaldehyde (pfa) and stored in 100% methanol at −20°C. In situ probes for the following genes were used: *hoxb1a*[63], *krox20*[64]*, pax2*[65]*, hoxb3a*[66]*, fgf3*[67]*,* and *hoxd4a*[68]. Visualization was completed using colorimetric reaction using NBT/BCIP or INT/BCIP in 10% polyvinyl alcohol. Embryos were dissected from the yolk and flat mounted in 70% glycerol for imaging on bridged coverslips as described (Zannino and Appel, 2009). Images were captured using a Nikon Eclipse E600 microscope equipped with Spot RT Color camera (model 2.2.1). Antibody labeling with mouse anti-Isl (39.4D5, 1:100; Developmental Studies Hybridoma Bank (DSHB)) and mouse anti-3A10 (1:100; DSHB) was visualized using the Alexa Fluro secondary antibody 488 goat anti-mouse (1:200; Molecular Probes). Embryos were fixed in 4% AB fix (4% paraformaldehyde, 8% sucrose, 1x PBS) for 2 hours at room temperature (RT) or overnight at 4C. Whole-mount fluorescent labeling was performed as described [69]. Embryos were imaged by an upright Zeiss Imager.M2 equipped with a 20x water immersion objective [numerical aperture (NA) = 1.0], mounted on a Nano-Drive, and a Perkin Elmer Ultraview system. Images were imported into Adobe Photoshop and adjustments were limited to contrast, levels, and cropping and were applied to the entire image.

### Micrococcal nuclease digestions and nucleosome identification

Micrococcal nuclease (MNase) digestions, isolation of mono-nucleosome fragments, and amplification of purified DNA was performed on 4 and 9 hour post fertilization embryos as previously published [46]. Nucleosomes were mapped to the *hoxb1a* promoter using a reported nucleosome scanning protocol [47]. Briefly, chromatin was isolated from embryos at 4hpf and 9hpf and digested with MNase. Mono-nucleosome sized fragments were purified and amplified by quantitative polymerase chain reaction (qPCR) using tiled primers spaced ~50 bp apart (See Additional file 6: Table S3 for primer sequences) from ~450 bp upstream to ~230 bp downstream of the *hoxb1a* TSS. qPCR was done using the Qiagen QuantiFast SYBR Green PCR Kit (Qiagen: 204054) on the ABI 7900HT Sequence detection system in a 384 well format and analyzed using SDS software v2.3. The signal from three biological replicates was averaged and values were expressed as a log_2_ ratio of the MNase sample to a control sample consisting of gDNA randomly fragmented by sonication. Statistical analysis was done using Student’s *t*-test in Microsoft Excel with significance cut-off set to p = 0.05.

## Competing interests

The authors declare that they have no competing interests.

## Authors’ contributions

SEW participated in the design of the study, generated the germ-line mutants, analyzed their phenotype and drafted the manuscript. AG participated in the design of the ZFNs and TALENs and assisted in the assessment of their activity. DAZ participated in the phenotypic analysis of the mutants. SAW participated in the design of the study, the design of the ZFNs and TALENs and the assessment of their function. CGS conceived of the study, participated in its design, coordinated the study and finalized the manuscript. All authors read, revised and approved the final manuscript.

## Supplementary Material

Additional file 1: Table S1 Sequences of two alleles with in-frame mutations. Click here for file

Additional file 2: Figure S1 Alignment of mutant Hoxb1a and Hoxb1b proteins. Peptide alignment based on conceptual translation of mutant *hoxb1a* (A) and *hoxb1b* (B) alleles. Red residues represent missense residues caused by the frameshift mutation prior to encountering a premature stop codon. Blue indicates homeodomain. Click here for file

Additional file 3: Table S2 Gene expression analysis of *hoxb1a* and *hoxb1b* mutants. Click here for file

Additional file 4: Figure S2 Crosses of multiple *hoxb1b* mutant alleles reveal a consistent hindbrain segmentation phenotype. 22hpf wild type (A, C, E, G, I, K) and *hoxb1b*^*−/−*^ (B, D, F, H, J, L) embryos were assayed by in situ hybridization for expression of *hoxb1a* in r4 (blue stain in panels A-L) and *krox20* in r3/r5 (red stain in panels A-L). All embryos are flat mounted in dorsal view with anterior to the top. Click here for file

Additional file 5: Figure S3 Crosses of multiple *hoxb1a* mutant alleles reveal a consistent hindbrain specification phenotype. 22hpf wild type (A, C, E) and *hoxb1a*^*−/−*^ (B, D, F) embryos were assayed by in situ hybridization for expression of *hoxb1a* in r4 (blue stain in panels A-F) and *krox20* in r3/r5 (red stain in panels A-F). All embryos are flat mounted in dorsal view with anterior to the top. Click here for file

Additional file 6: Table S3 Primers used for genotyping and nucleosome scanning. Click here for file
