# Supplementary material for: Targeted germ line disruptions reveal general and species-specific roles for paralog group 1 hox genes in zebrafish
Source: BMC Dev Biol. 2014 Jun 5;14:25. doi: 10.1186/1471-213X-14-25 (PMC4061917; doi:10.1186/1471-213X-14-25)
Supplement: Additional file 1: Table S1 — Sequences of two alleles with in-frame mutations. [file 1471-213X-14-25-S1.pdf]

Table S1. Sequences of two alleles with in-frame mutations.

Founder	Nuclease	Sequence[a]	Type of mutation[b]
hoxb1a		TTTGTAA**CCGTGG**GACGA	
A12	Tb1a-2	TTTGTAACGCCTACAATTTAGGGACGA	Insertion (+12bp)
hoxb1b		GCCCTTCCA**CCTCAACGTGG**ACATGGG	
B1	Zb1b-3	GCCCTT GGACATGGG	Deletion (-12bp)

[a] Sequence of mutant alleles. Deletions are shown as gaps and insertions are indicated in red. Bold and underlined nucleotides indicate site for diagnostic restriction enzyme in wild type sequence.
[b] Indicates whether mutation results from insertion, deletion or both. Number in parenthesis indicates net gain/loss of nucleotides in mutant sequence.
