# Supplementary material for: Targeted germ line disruptions reveal general and species-specific roles for paralog group 1 hox genes in zebrafish
Source: BMC Dev Biol. 2014 Jun 5;14:25. doi: 10.1186/1471-213X-14-25 (PMC4061917; doi:10.1186/1471-213X-14-25)
Supplement: Additional file 3: Table S2 — Gene expression analysis of hoxb1a and hoxb1b mutants. [file 1471-213X-14-25-S3.pdf]

Table S2[a]. Gene expression phenotypes of *hoxb1a* and *hoxb1b* mutants.

Cross	Genotype	Gene Expression Phenotype											
		hoxb1a		*hoxb3a*		*hoxd4a*		*krox20*		*pax2*		*fgf3*	
		WT	MUT	WT	MUT	WT	MUT	WT	MUT	WT	MUT	WT	MUT
$hoxb1a^{+/um189}$ x $hoxb1a^{+/um190}$	$b1a^{+/+}$, $b1a^{+/-}$, $b1a^{-/+}$	39	0					39	0				
	$b1a^{-/-}$	0	11					11	0				
$hoxb1a^{+/um191}$ x $hoxb1a^{+/um192}$	$b1a^{+/+}$, $b1a^{+/-}$, $b1a^{-/+}$	43	0	38	0	39	0	43	0	39	0		
	$b1a^{-/-}$	0	12	9	0	9	0	12	0	9	0		
$hoxb1b^{+/um195}$ x $hoxb1b^{+/um195}$	$b1b^{+/+}$, $b1b^{+/-}$, $b1b^{-/+}$	40	0					40	0				
	$b1b^{-/-}$	3	16					3	16				
$hoxb1b^{+/um196}$ x $hoxb1b^{+/um196}$	$b1b^{+/+}$, $b1b^{+/-}$, $b1b^{-/+}$	36	0					36	0				
	$b1b^{-/-}$	0	10					0	10				
$hoxb1b^{+/um197}$ x $hoxb1b^{+/um197}$	$b1b^{+/+}$, $b1b^{+/-}$, $b1b^{-/+}$	35	0	45	1	38	0	35	0	38	0		
	$b1b^{-/-}$	0	12	1	9	14	0	0	12	14	0		
$hoxb1b^{+/um195}$ x $hoxb1b^{+/um196}$	$b1b^{+/+}$, $b1b^{+/-}$, $b1b^{-/+}$	33	1					33	1				
	$b1b^{-/-}$	1	12					1	12				
$hoxb1b^{+/um195}$ x $hoxb1b^{+/um197}$	$b1b^{+/+}$, $b1b^{+/-}$, $b1b^{-/+}$	39	4					39	4				
	$b1b^{-/-}$	0	11					0	11				
$hoxb1b^{+/um196}$ x $hoxb1b^{+/um197}$	$b1b^{+/+}$, $b1b^{+/-}$, $b1b^{-/+}$	34	0					34	0				
	$b1b^{-/-}$	0	11					0	11				
$hoxb1a^{+/um193};hoxb1b^{+/um197}$ x $hoxb1a^{+/um194};hoxb1b^{+/um197}$	$b1a^{+/+};b1b^{+/+}$, $b1a^{+/+};b1b^{+/-}$, $b1a^{+/+};b1b^{-/+}$, $b1a^{+/-};b1b^{+/+}$, $b1a^{+/-};b1b^{+/-}$, $b1a^{+/-};b1b^{-/+}$, $b1a^{-/+};b1b^{+/+}$, $b1a^{-/+};b1b^{+/-}$, $b1a^{-/+};b1b^{-/+}$	111	1			106	0	216	2	106	0	64	1
	$b1a^{+/+};b1b^{-/-}$, $b1a^{-/+};b1b^{-/-}$, $b1a^{+/-};b1b^{-/-}$	0	41			29	0	5	65	29	0	0	12
	$b1a^{-/-};b1b^{+/+}$, $b1a^{-/-};b1b^{+/-}$, $b1a^{-/-};b1b^{-/+}$	0	25			19	0	44	0	19	0	2	17
	$b1a^{-/-};b1b^{-/-}$	0	9			5	0	0	14	5	0	0	7

[a]Embryos from the indicated crosses were assayed by in situ hybridization for expression of the genes listed. Expression was scored as wild type (WT) if gene expression was normal and as mutant (MUT) if expression level or size of expression domain were altered (see Fig. 2 for examples of mutant phenotypes). Embryos were grouped based on phenotype and subsequently genotyped.
