# Supplementary material for: Targeted germ line disruptions reveal general and species-specific roles for paralog group 1 hox genes in zebrafish
Source: BMC Dev Biol. 2014 Jun 5;14:25. doi: 10.1186/1471-213X-14-25 (PMC4061917; doi:10.1186/1471-213X-14-25)
Supplement: Additional file 4: Figure S2 — Crosses of multiple hoxb1b mutant alleles reveal a consistent hindbrain segmentation phenotype. 22hpf wild type (A, C, E, G, I, K) and hoxb1b−/− (B, D, F, H, J, L) embryos were assayed by in situ hybridization for expression of hoxb1a in r4 (blue stain in panels A-L) and krox20 in r3/r5 (red stain in panels A-L). All embryos are flat mounted in dorsal view with anterior to the top. [file 1471-213X-14-25-S4.pdf]

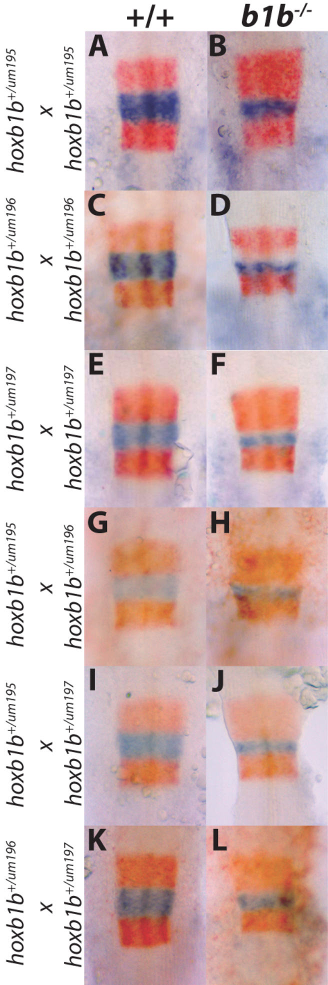

+/+
b1b-/-
A
B
C
D
E
F
G
H
I
J
K
L
hoxb1b+/um195 x hoxb1b+/um195
hoxb1b+/um196 x hoxb1b+/um196
hoxb1b+/um197 x hoxb1b+/um197
hoxb1b+/um195 x hoxb1b+/um196
hoxb1b+/um195 x hoxb1b+/um197
hoxb1b+/um196 x hoxb1b+/um197
