# Supplementary figures and images for: Targeted germ line disruptions reveal general and species-specific roles for paralog group 1 hox genes in zebrafish
Source: BMC Dev Biol. 2014 Jun 5;14:25. doi: 10.1186/1471-213X-14-25 (PMC4061917; doi:10.1186/1471-213X-14-25)

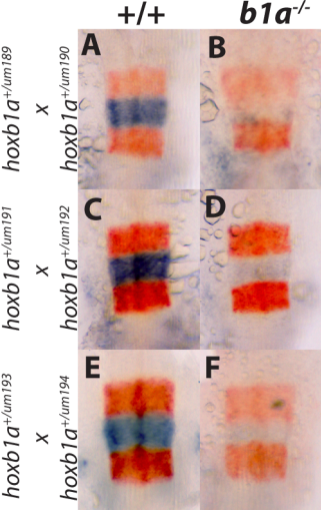

Supplement: Additional file 5: Figure S3 — Crosses of multiple hoxb1a mutant alleles reveal a consistent hindbrain specification phenotype. 22hpf wild type (A, C, E) and hoxb1a−/− (B, D, F) embryos were assayed by in situ hybridization for expression of hoxb1a in r4 (blue stain in panels A-F) and krox20 in r3/r5 (red stain in panels A-F). All embryos are flat mounted in dorsal view with anterior to the top. [file 1471-213X-14-25-S5.pdf]
