# Supplementary material for: Targeted germ line disruptions reveal general and species-specific roles for paralog group 1 hox genes in zebrafish
Source: BMC Dev Biol. 2014 Jun 5;14:25. doi: 10.1186/1471-213X-14-25 (PMC4061917; doi:10.1186/1471-213X-14-25)
Supplement: Additional file 6: Table S3 — Primers used for genotyping and nucleosome scanning. [file 1471-213X-14-25-S6.pdf]

Table S3. Primers used for genotyping and nucleosome scanning.

Primer Name	Primer Sequence	Primer Purpose
hoxb1a TALEN FWD	TTTCTCAGGTTGTCCCTCCG	Identify *hoxb1a* TALEN lesion
hoxb1a TALEN RVS	TTATAGCTGTCACTAGCGTGTCC	Identify *hoxb1a* TALEN lesion
hoxb1b ZFN FWD	CACCGCACGAAACTCATGGC	Identify *hoxb1b* ZFN lesion
hoxb1b ZFN RVS	AATGAGGAGGTCTGGTTTGCTTGC	Identify *hoxb1b* ZFN lesion
hoxb1bum197 FWD	CACAAATTCAATCGTGTTTCAATCGTG	Identify *hoxb1b*^{um197} lesion (used with *hoxb1b* ZFN RVS)
NS Fragment 1 FWD	CAGATTTCCTTCCTAAACACACA	Nucleosome scanning (NS)
NS Fragment 1 RVS	ATTAAAGAGGACAATCTAGCTCACA	Nucleosome scanning (NS)
NS Fragment 2 FWD	GGTAAACGCGAACATTACTCC	Nucleosome scanning (NS)
NS Fragment 2 RVS	GGAAGATAGCACATTCGTAATTAAA	Nucleosome scanning (NS)
NS Fragment 3 FWD	CCTATGCTCCAGTCCATTACG	Nucleosome scanning (NS)
NS Fragment 3 RVS	GGTTAAAAGATGCAAGGGGA	Nucleosome scanning (NS)
NS Fragment 4 FWD	GGTGCGATTAAAATTAGAAACTAATGG	Nucleosome scanning (NS)
NS Fragment 4 RVS	AATGAGAGAAAAAGAAATAAAGAAAGAGCGC	Nucleosome scanning (NS)
NS Fragment 5 FWD	AAAGATGCAAGGGGATGAAG	Nucleosome scanning (NS)
NS Fragment 5 RVS	TATGTCAAACCCTGCGTGAAAGG	Nucleosome scanning (NS)
NS Fragment 6 FWD	AAGCGCTCTTTCTTTATTTCTTTTTCTCTC	Nucleosome scanning (NS)
NS Fragment 6 RVS	AAAGCCACTTCAATCAAACCAGCC	Nucleosome scanning (NS)
NS Fragment 7 FWD	TTTCACGCAGGGTTTGAC	Nucleosome scanning (NS)
NS Fragment 7 RVs	AAGTTTGTCAGCGCACGGC	Nucleosome scanning (NS)
NS Fragment 8 FWD	TTTGATTGAAGTGGCTTTGTCATGC	Nucleosome scanning (NS))
NS Fragment 8 RVS	TGAGACGTCACGGCGCC	Nucleosome scanning (NS)
NS Fragment 9 FWD	TGACAAACTTCTGGAGGTCCCC	Nucleosome scanning (NS)

NS Fragment 9 RVS	TTACCTCTGGAGTATTTGCTCGTGC	Nucleosome scanning (NS))
NS Fragment 10 FWD	CCAGCAGCTGAGGTAAAGATG	Nucleosome scanning (NS)
NS Fragment 10 RVS	CTTCCGCATGACATACTATTGC	Nucleosome scanning (NS)
NS Fragment 11 FWD	AAGCACGAGCAAATACTCCAGAGG	Nucleosome scanning (NS)
NS Fragment 11 RVS	AATTAATGGCGGAGGGACAACC	Nucleosome scanning (NS)
NS Fragment 12 FWD	ATTGCGAGCTTACAGGACAGGAGG	Nucleosome scanning (NS)
NS Fragment 12 RVS	TTCGTCCCACGGTTACAAATTGTG	Nucleosome scanning (NS)
NS Fragment 13 FWD	TTCTCAGGTTGTCCCTCCGCC	Nucleosome scanning (NS)
NS Fragment 13 RVS	AAGTGGTGGTATCCAGCCTTGG	Nucleosome scanning (NS)
NS Fragment 14 FWD	ATTTGTAACCGTGGGACGAA	Nucleosome scanning (NS))
NS Fragment 14 RVS	CTGGACACGCTAGTGACAGC	Nucleosome scanning (NS)
NS Fragment 15 FWD	TTGGACCAGGCGTTCCCG	Nucleosome scanning (NS))
NS Fragment 15 RVS	TTCTGGTGCTGATGTTGTGCTGC	Nucleosome scanning (NS)
NS Fragment 16 FWD	TCCACACTGGACACGCTAGT	Nucleosome scanning (NS)
NS Fragment 16 RVS	TTCTGGTGCTGATGTTGTGC	Nucleosome scanning (NS)
NS Fragment 17 FWD	AATCAGCCACCAACAGCAGC	Nucleosome scanning (NS)
NS Fragment 17 RVS	TTTGATTTTGGTGCTGGTGATGC	Nucleosome scanning (NS))
NS Fragment 18 FWD	AACATCAGCACCAGAACGGC	Nucleosome scanning (NS))
NS Fragment 18 RVS	ATAACTTGTTGTCCCAGTTCCACC	Nucleosome scanning (NS))
