# Supplementary material for: Targeted germ line disruptions reveal general and species-specific roles for paralog group 1 hox genes in zebrafish
Source: BMC Dev Biol. 2014 Jun 5;14:25. doi: 10.1186/1471-213X-14-25 (PMC4061917; doi:10.1186/1471-213X-14-25)
Supplement: Additional file 2: Figure S1 — Alignment of mutant Hoxb1a and Hoxb1b proteins. Peptide alignment based on conceptual translation of mutant hoxb1a (A) and hoxb1b (B) alleles. Red residues represent missense residues caused by the frameshift mutation prior to encountering a premature stop codon. Blue indicates homeodomain. [file 1471-213X-14-25-S2.pdf]

A

```
               ************** :
hoxb1a_WT      MDSSRMNSFLEYTICNRGTNAYSPKAGYHHLDQAFPGPFHTGHASDSYNADGRLYVGGSNQPPTAAAQHQHQNGIYAHHQ   80
hoxb1a_um189   MDSSRMNSFLEYTICNVGRTPTRPRLDTTTWTRRSRALSTLDTLVTAITLMDDFT   55
hoxb1a_um190   MDSSRMNSFLEYTICTQFVQFGRTPTRPRLDTTTWTRRSRALSTLDTLVTAITLMDDFT   59
hoxb1a_um191   MDSSRMNSFLEYTICTDERLLAQGWIPPLGPGVPGPFPHWTR   42
hoxb1a_um192   MDSSRMNSFLEYTICTPFVLLAQGWIPPLGPGVPGPFPHWTR   42
hoxb1a_um193   MDSSRMNSFLEYTIWDERLLAQGWIPPLGPGVPGPFPHWTR   41
hoxb1a_um194   MDSSRMNSFLEYTICNLDERLLAQGWIPPLGPGVPGPFPHWTR   43

hoxb1a_WT      HQNQTGMGLTYGGTGTTSYGTQACANSDYAQHQYFINPEQDGMYYHSSGFSTSNASPHYGSMAGAYCGAQGAVPAAPYQH   160
hoxb1a_um189   55
hoxb1a_um190   59
hoxb1a_um191   42
hoxb1a_um192   42
hoxb1a_um193   41
hoxb1a_um194   43

hoxb1a_WT      HGCEGQDHQRAYSQGTYADLSASQGTEKDTDQPPPGKTFDWMKVKRNPPKTGKVAEYGLGPQNTIRTNFTTKQLTELEKE   240
hoxb1a_um189   55
hoxb1a_um190   59
hoxb1a_um191   42
hoxb1a_um192   42
hoxb1a_um193   41
hoxb1a_um194   43

hoxb1a_WT      FHFSKYLTRARRVEIAATLELNETQVKIWFQNRRMKQKKREKEGLAPASSTSSKDLEDQSDHSTSTSPEASPSPDS   316
hoxb1a_um189   55
hoxb1a_um190   59
hoxb1a_um191   42
hoxb1a_um192   42
hoxb1a_um193   41
hoxb1a_um194   43
```

B

```
               **********************************************************************   :
hoxb1b_WT      MNSYLDYTIYNRGSNTYSSKVGCFPVEQEYLPSACASTNSYIPEGRPVGGNTFTSAPHETHGTSYAQIQSQPFHLNVDMG   80
hoxb1b_um195   MNSYLDYTIYNRGSNTYSSKVGCFPVEQEYLPSACASTNSYIPEGRPVGGNTFTSAPHETHGTSYAQIQSQPFRGHG   77
hoxb1b_um196   MNSYLDYTIYNRGSNTYSSKVGCFPVEQEYLPSACASTNSYIPEGRPVGGNTFTSAPHETHGTSYAQIQSQPFHIPWTWV   80
hoxb1b_um197   MNSYLDYTIYNRGSNTYSSKVGCFPVEQEYLPSACASTNSYIPEGRPVGGNTFTSAPHETHGTSYAQIQSCFNRETQIHK   80

hoxb1b_WT      KTGHSNFCKQTRPPHSDYGHQHVLTQADDHMRLQSPGFSVVNMGANIGTYSESNCRPGSVSASHYQSYAYGEPEPHGYGS   160
hoxb1b_um195   77
hoxb1b_um196   KQDTAISASKPDLLIRTMDINTFSLKQMTTCAFSPLAFPS   120
hoxb1b_um197   FNRGHG   86

hoxb1b_WT      FSKYQVSPDSDSDSKTNIKQAPTFDWMKVKRNPPKTVKVAEYGIHGQQNIIRTNFTTKQLTELEKEFHFNKYLTRARRVE   240
hoxb1b_um195   77
hoxb1b_um196   120
hoxb1b_um197   86

hoxb1b_WT      VAATLELNETQVKIWFQNRRMKQKKREKEGTAPVIKRVTLCSSGQNADHSTSSSPGASPTSDSSTAI   307
hoxb1b_um195   77
hoxb1b_um196   120
hoxb1b_um197   86
```
